# Unsupervised Low-Light Image Enhancement Based on Generative Adversarial Network

**DOI:** 10.3390/e25060932

**Published:** 2023-06-13

**Authors:** Wenshuo Yu, Liquan Zhao, Tie Zhong

**Affiliations:** Key Laboratory of Modern Power System Simulation and Control & Renewable Energy Technology, Ministry of Education, Northeast Electric Power University, Jilin 132012, China; 2202100367@neepu.edu.cn (W.Y.); zhaoliquan@neepu.edu.cn (L.Z.)

**Keywords:** generative adversarial networks, low-light image enhancement, hybrid attention module, parallel dilated convolution module

## Abstract

Low-light image enhancement aims to improve the perceptual quality of images captured under low-light conditions. This paper proposes a novel generative adversarial network to enhance low-light image quality. Firstly, it designs a generator consisting of residual modules with hybrid attention modules and parallel dilated convolution modules. The residual module is designed to prevent gradient explosion during training and to avoid feature information loss. The hybrid attention module is designed to make the network pay more attention to useful features. A parallel dilated convolution module is designed to increase the receptive field and capture multi-scale information. Additionally, a skip connection is utilized to fuse shallow features with deep features to extract more effective features. Secondly, a discriminator is designed to improve the discrimination ability. Finally, an improved loss function is proposed by incorporating pixel loss to effectively recover detailed information. The proposed method demonstrates superior performance in enhancing low-light images compared to seven other methods.

## 1. Introduction

Images captured in low-light conditions, such as cloudy days and nights, exhibit low brightness, high noise levels, and color distortion. These factors significantly impact the accuracy of high-level vision tasks, including object detection [1] and image segmentation [2]. Therefore, enhancing low-light images becomes crucial for improving the accuracy of such tasks under low-light conditions. Traditional low-light image enhancement methods primarily rely on histogram equalization and Retinex theory. The histogram equalization method enhances the global contrast of the image by stretching its histogram distribution. However, it tends to amplify background noise while reducing the contrast of useful information. Additionally, it can cause local oversaturation and loss of detailed information [3]. On the other hand, the Retinex theory method estimates the illumination component from the original image and removes it to obtain an enhanced image. However, it may introduce a halo phenomenon, especially in images with significant lighting variations, which affects the visual quality of the image [4]. With the advancements in deep-learning techniques, low-light image enhancement methods based on deep learning have been proposed. These methods utilize paired image data to train the network and to obtain a model that can enhance low-light images. These methods require paired images that are low-light images and original high-definition images. However, acquiring paired images in real-world scenarios is challenging. Consequently, unsupervised low-light image enhancement methods based on generative adversarial networks (GANs) have been proposed [5,6]. These methods do not rely on paired images for training and are better suited for practical applications.

To enhance the quality of enhanced images, we propose a generative adversarial network (GAN) for unsupervised low-light image enhancement. The network comprises a generator and a discriminator. The generator is responsible for generating high-quality images, while the discriminator determines whether an image is a generated high-quality image or an original high-quality image. By leveraging the interplay between the generator and discriminator, we enhance the image generation capability of the generative network. The parameters of both networks are adjusted based on the output of the discriminator. Through iterative training, the adversarial network becomes unable to distinguish between generated and original images. Upon completion of training, we utilize the trained generator to enhance low-light images.

The main contributions of this paper are summarized as follows:

- We designed a novel generator for enhancing low-light images. Firstly, we designed a hybrid attention module consisting of a channel attention module and a pixel attention module. Secondly, we utilized the hybrid attention module as a sub-module within the design of the residual module. Thirdly, we designed a parallel dilated convolution module to capture multiscale information. Lastly, we combined the designed residual module with the hybrid attention module, and parallel dilated convolution module to construct the generative network. Additionally, we employed a skip connection to fuse shallow features with deep features, enhancing the representation of the generative network.
- We propose an adversarial network that includes two discriminators: a global discriminator and a local discriminator. The local discriminator is constructed using six standard convolution layers, while the global discriminator employs three different dilated convolution layers, skip connections, and four standard convolution layers.
- We propose an improved loss function by introducing pixel loss into the loss function of the generative adversarial network. It is beneficial for recovering detailed image information

In this section, we introduce the background of low-light image enhancement and our contribution, and Section 2 presents the related work on low-light image enhancement. In Section 3, we explain our proposed method in detail. In Section 4, we show and compare our simulation results. In Section 5, the conclusion is given.

## 2. Related Work

In recent years, numerous methods have been proposed for low-light image enhancement. These methods can be categorized into traditional techniques and deep-learning approaches. The traditional methods can be further divided into two categories: low-light image enhancement based on histogram equalization and low-light image enhancement based on Retinex theory. The low-light image enhancement method based on histogram equalization aims to improve the image by adjusting the distribution of pixel intensities in the histogram, thereby altering the grayscale of each pixel. However, images enhanced using this method often suffer from significant noise, color distortion, and loss of detailed information [7]. The low-light image enhancement method based on Retinex theory enhances low-quality images by estimating the illumination component within the image and then subtracting this estimated component from the original image. Based on Retinex theory, the interference of noise in the reflectance component is not considered, thus leading to artifacts and overexposure in the enhanced image [8].

With the advancement of deep-learning techniques, a multitude of low-light image enhancement methods have been developed based on deep-learning approaches. These methods have shown superior performance in enhancing image quality compared to traditional methods. For instance, Wei et al. introduced a data-driven Retinex-Net method [9]. Firstly, the proposed method decomposed the input image into illumination and reflectance components. To address the issue of noise interference in the reflectance component, the BM3D algorithm was employed, effectively mitigating the noise interference in the Retinex-Net theory-based method. Secondly, an encoder-decoder network was utilized to extract feature information from the illumination component, aiming to alleviate the impact of lighting variations on the image. Lastly, the adjusted illumination and reflectance components were combined to enhance the low-light image. However, this method cannot effectively recover the color information of the image. Yang et al. introduced a deep recursive band network as a semi-supervised network for enhancing low-light images [10]. The network consists of two stages: band recomposition and recursive band learning. In the band recomposition stage, the network is trained using paired low-light and normal-light images. The trained network reconstructs a linear band representation of an enhanced normal-light image. In the recursive band learning stage, learnable linear transformations and given bands are utilized to recover the color information of the image. While the enhanced image produced by this method exhibits improved color distributions, visually appealing contrast, and preserved detailed information, it is important to note that it does not effectively eliminate the presence of noise. Guo et al. introduced a method called Zero-reference deep curve estimation for low-light image enhancement [11]. This approach utilizes a deep convolutional network to estimate the dynamic range of the higher-order curve and applies pixel-level adjustments to generate the enhanced image. One significant advantage of this method is that it does not rely on paired images, thus avoiding potential degradation of image quality caused by overfitting. However, the method has a large number of image parameters, and the training and inference time is longer. Li et al. introduced the Zero-DCE++ method [12], which is based on the Zero-DCE approach. In Zero-DCE++, convolution layers in Zero-DCE are replaced with depthwise separable convolutions, reducing the overall number of network parameters. This modification effectively addresses the issues associated with the large parameter count and lengthy training time of the original Zero-DCE method. However, it is important to note that the Zero-DCE++ method may introduce an overexposure phenomenon in the enhanced images.

To further enhance the quality of the enhanced image, generative adversarial networks have been employed in low-light image enhancement. The generative adversarial networks are a special type of deep-learning model that has found wide applications in various tasks such as image denoising [13], image super-resolution [14,15], and image classification [16]. In recent years, researchers have also applied generative adversarial networks in the domain of image enhancement. Hua et al. introduced a method for joint image quality assessment using generative adversarial networks [17]. In their approach, they utilized a multi-term perceptual loss function that incorporated image quality assessment, content, and texture within the generative adversarial network. This combination of loss terms helped in reducing the presence of artifacts in the enhanced image. However, it should be noted that this method may result in a loss of certain color feature information. Kim et al. proposed a low-light GAN method [18]. In their approach, they incorporated spectral normalization and a color loss function to enhance the efficiency of network training and to improve the extraction of color information. Additionally, they employed local illumination during the training process to address saturation issues in bright areas. However, it is worth mentioning that the method encountered challenges associated with underexposure. Shi et al. introduced a low-light image enhancement method that combines Retinex theory with a generative adversarial network [19]. Their approach involves employing the U-Net network as both the decomposition and enhancement modules within the generative network. To address the issue of image blurring, the method incorporated a structural similarity loss. However, it is important to note that the method ignored the impact of noise in the enhanced images. Guo et al. proposed a multiscale feature-guided low-light image enhancement method [20]. The method employed a generative adversarial network as the baseline, and a multiscale feature-guided attention mechanism was integrated into the generator. This attention mechanism aided in removing noise information from the image, resulting in improved performance for non-uniform-light image enhancement. However, it should be noted that the enhanced images produced by this method exhibited color deviation issues.

Yang et al. introduced a generative adversarial network method based on Vision Transformer (VIT) [21]. The method involved two branches. The first branch employed an iterative multi-branch network for feature extraction, while the second branch utilized a reconstruction module for image enhancement. By combining the VIT generator, which was designed based on multi-head multi-channel attention (MHMCA) and local feature fusion module (LFFM), with a conventional convolutional discriminator, the method effectively addressed challenges such as chromatic aberration, artifacts, and noise commonly encountered in low-light image enhancement. However, it is worth noting that the method has a large number of parameters, which can contribute to longer training times. Jiang et al. introduced the EnlightenGAN method [22]. Their approach utilized a self-feature-preserving loss and a self-regularized attention mechanism for unsupervised low-light image enhancement. However, the enhanced images by this method often exhibit obvious artifacts. Li et al. proposed an effective generative adversarial network method [23]. This method designed a dense residual block and an enhancement block for low-light image enhancement, successfully mitigating the presence of artifacts in the images. Nonetheless, the method may face challenges when enhancing images with uneven illumination. Qu et al. presented a generative adversarial network with a multiple discriminators method [24]. The method employed a multiscale discriminator to evaluate images from different perspectives and utilized a feature fusion attention module to address issues related to uneven illumination in images. However, it is important to note that the method does not explicitly consider the impact of noise. Rao et al. introduced a component enhancement network based on a generative adversarial network and Retinex theory [25]. Their network used a parallel two-branch structure to enhance both the illumination and reflectance components, effectively mitigating the interference of noise.

Although the enhanced image quality is better using the generative adversarial network-based method, there are still large differences between the enhanced image and the real normal-light image, especially since the enhanced image has noise and artifacts, and some regions are overexposed or underexposed. Such issues severely impact the overall quality of the enhanced images. To address these limitations and further enhance the quality of the results, we propose a novel generative adversarial network in this paper. Experimental results demonstrate that our method effectively improves the quality of the enhanced images.

## 3. Proposed Methods

The mathematical model of low-light image enhancement based on a deep-learning module can be expressed as follows:

(1) $$\hat{R}=F\left(P;\theta \right)$$

where $P\in {\mathbb{R}}^{3\times H\times W}$ represents the low-light image with height H and width W, θ are the parameters of the deep-learning module, F() is a deep-learning model, and $\hat{R}\in {\mathbb{R}}^{3\times H\times W}$ represents the enhanced image obtained from the low-light image P using the deep-learning model F(). The loss function is used to measure the distance between the enhanced image and the normal-light image. By employing the gradient descent method, the parameters of the deep-learning model are iteratively optimized to minimize the loss function and obtain the optimal network parameters. The optimal network parameters are given by:

(2) $$\hat{\theta }=\underset{\theta }{argminLoss}\left(\hat{R},G\right)$$

where $G\in {\mathbb{R}}^{3\times H\times W}$ represents the normal-light image.

Based on (1), we designed a novel generative adversarial network to improve the quality of enhanced images derived from low-light images in an unsupervised manner. Our generative adversarial network comprises a generator and a discriminator. The generator is responsible for enhancing the low-light image. It takes the original low-light image as input and generates an enhanced image. The discriminator, on the other hand, serves to determine whether the input image is a generated high-quality image or the original high-quality image. The output of the generator, as well as the original high-quality image, are used as the inputs for the discriminator. The original high-quality image and original low-light image are not paired images. To train our network, we employ a loss function that measures the performances of both the generator and the discriminator. The loss function guides the optimization process using the Adam optimizer to achieve optimal performance for low-light image enhancement. Through multiple iterations of training, the discriminator becomes incapable of distinguishing between high-quality images and generated images from low-light images. After the training, we use the trained generator to enhance the low-light image.

### 3.1. Proposed Generator

**Figure 1:**
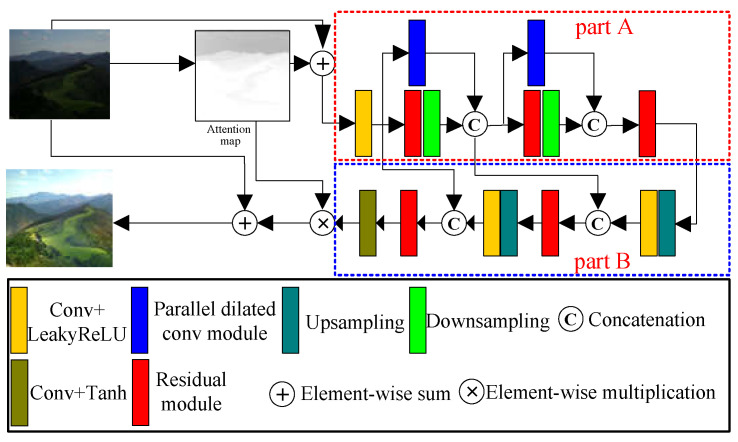
Proposed generator. (**Part A**) the top-down network, (**Part B**) the bottom-up network.

We designed a generator for enhancing low-light images without supervision. The architecture of our proposed generator is shown in Figure 1. Our generator adopted the traditional encoder-decoder structure, which has been proven to be effective in the field of image enhancement through a large number of experiments. The generator comprises two main components: the top-down network (Part A in Figure 1) and the bottom-up network (Part B in Figure 1). In the top-down network, we employed downsampling operations to reduce the feature map size, thereby increasing the receptive field of the network. This can extract more low-frequency information that captures the general outline information of the image. Conversely, the bottom-up network utilizes upsampling operations to enlarge the feature map size, allowing the network to focus on high-frequency information that contains more detailed image features. To effectively fuse the high-frequency and low-frequency information, we incorporated short connections that connect the top-down and bottom-up networks. These connections facilitate the exchange of information between different levels of the network and enhance the integration of features. The overall architecture of our generative network is depicted in Figure 1.

Inspired by Jiang et al. [22], we performed a preprocessing operation on the original low-light image to prevent overexposure and underexposure of the output image. Firstly, we utilized the luminosity method to convert the original RGB image into a one-channel grayscale map, denoted as I. Secondly, we normalized the grayscale map to the range [0, 1], enabling us to obtain the luminance weight value of each pixel in the image. Finally, we used the image obtained by 1-I as our attention map. This attention map assigns larger weight values to pixels in the dark areas, while smaller weight values are assigned to pixels in the light areas. This approach allows the network to focus more on enhancing the dark areas of the image. The low-light image is element-wise summed with its attention map, and the output is used as the input of the generative network. In our designed generative network, we first used a standard convolution and LeakyReLU activation function to increase the number of channels from 3 to 32. To extract more useful features, we designed a residual module with a hybrid attention module, which further increased the number of channels from 32 to 64. The input of the designed residual module is the output of the first LeakyReLU activation.

To extract low-frequency information, we increased the receptive field of the network by downsampling the output of the residual module. The size of the output feature map obtained by downsampling is half the size of the input feature map. To extract features at different scales and depths, we designed a parallel dilated convolution module. This module shares the same input feature map and number of output channels as the residual module. The size of the output feature map and the number of output channels are equivalent to those of the first downsampling layer. The output of the parallel dilated convolution module and the output of the first downsampling layer are connected by concatenation operation. For simplicity, we named the above module as module 1, which consists of our designed residual module, parallel dilated convolution module, and downsampling layer. To further extract low-frequency information, we constructed module 2 using the same parallel dilated convolution module, residual module, and downsampling layer as in module 1. Module 2 has an identical structure to module 1

The designed residual module was used to extract feature information from the output feature map of module 2. Subsequently, an upsampling layer and a standard convolution layer with the LeakyReLU activation function were applied to increase the feature map’s size and adjust the number of output channels. To reduce the loss of information during upsampling, we employed a concatenation operation to merge the output feature map of the LeakyReLU activation function with the out feature map of module 1. Next, we utilized our proposed residual module to extract detailed information from the fused feature map. Additionally, we still used the upsampling layer, standard convolution with LeakyReLU activation function, and short connection and residual module to fuse shallow features and extract more detailed information. To obtain the weight for each pixel, we used a standard convolution with a Tanh activation function to adjust the number of output channels to 3. Finally, by multiplying the weight with the attention map and adding it to the original low-light image, we obtained the enhanced image

**Figure 2:**
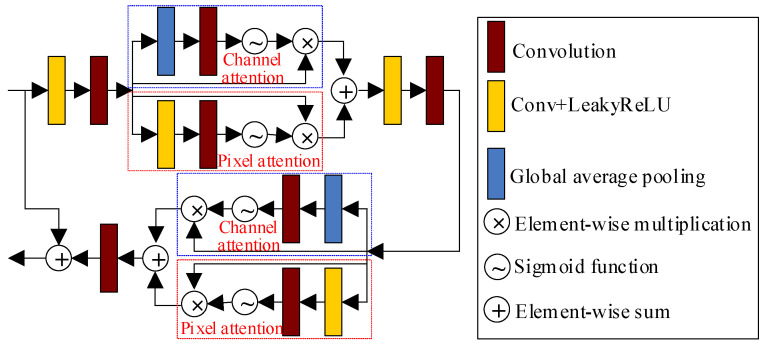
Proposed residual module with a hybrid attention mechanism.

In our designed generator, as shown in Figure 1, we designed a residual module with a hybrid attention module to extract features, as shown in Figure 2. This design effectively extracts the deep features while preventing issues such as gradient disappearance and gradient explosion. The designed residual module consists of two sets of hybrid attention modules, the convolution layer with LeakyReLU activation function, a standard convolution layer, and a skip connection. Such structure is more conducive to extracting more feature information and also improves the correlation between different information. The hybrid attention module, as shown in Figure 2, consists of a channel attention module and a pixel attention module. The channel attention module is composed of two branches. The first branch is a skip connection to retain input feature maps. The second branch computes the weight of different channels of feature maps. The more important the feature map, the larger the weight of the corresponding channel of the feature map. The final output feature maps of channel attention are obtained by multiplying the outputs of two branches. The second branch incorporates global average pooling, convolution layer, and sigmoid activation function. The global average pooling converts the global information into a 1×1×C (C is the number of channels) feature map. The convolution layer extracts features, and the sigmoid activation function determines feature weights.

The pixel attention module also consists of two branches. The first branch is also a skip connection to retain input feature maps. The second branch computes the weight of different pixels of feature maps, assigning larger weights to more important pixels. This branch includes a convolution layer with a LeakyReLU activation function, a standard convolution layer, and a sigmoid activation function. The convolution layer with the LeakyReLU activation function is responsible for extracting the pixel features from the feature map. The standard convolution layer and sigmoid activation function are used to extract features and determine pixel weights. The final output feature maps of pixel attention are obtained by multiplying the outputs of the two branches. Finally, the output feature map of the hybrid attention mechanism is obtained by element-wise summation of the output feature maps from the pixel attention module and the channel attention module.

The output of the hybrid attention module can be expressed as follows:

(3) F(x)=C(x)⊕P(x)

where x is the input feature map of the hybrid attention module and C(x) and P(x) are the output feature map of the channel attention module and pixel attention module, respectively. They are expressed as follows:

(4) $$C(x)=x⊗\sigma \{Conv_{2}[pool(x)]\}$$

(5) $$P(x)=x⊗\sigma \{Conv_{2}[Conv_{1}(x)]\}$$

where pool() represents the global average pooling operation. $Conv_{1}()$ represents the convolution layer with the LeakyReLU activation function. σ(⋅) represents the sigmoid activation function. $Conv_{2}()$ represents the convolution layer. The global average pooling operation $pool(x_{k})$ is expressed as

(6) $$pool(x_{k})=\frac{1}{W\times H}\sum_{i=1}^{W}\sum_{j=1}^{H}x_{k}(i,j)$$

where $x_{k}$ represents the feature map on the kth channel. W and H represent the width and height of the feature map, respectively.

**Figure 3:**
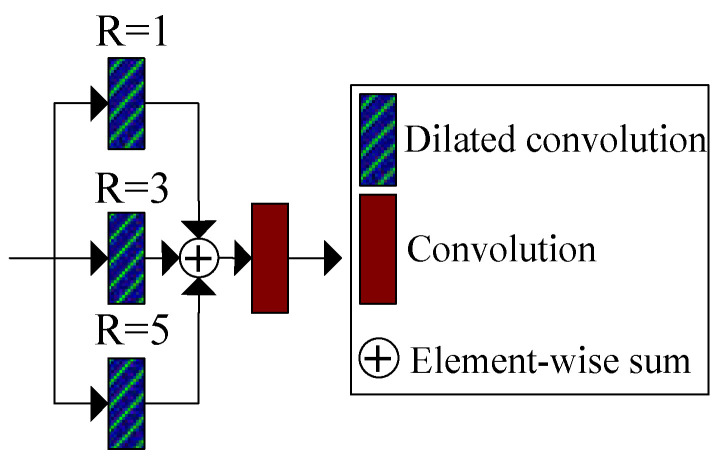
Proposed parallel dilated convolution module.

In the generator shown in Figure 1, the downsampling operation will lose some features. To reduce information loss and extract more low-frequency information with different scales, we designed a parallel dilated convolution module. This module comprises three parallel dilated convolutions with dilated rates of 1, 3, and 5, along with a standard convolution layer. Compared with standard convolution, dilated convolution can increase the receptive field with the same kernel size. This enables the extraction of more effective low-frequency information. The structure of the parallel dilated convolution module is shown in Figure 3.

For the generator, our proposed residual module and parallel dilated convolution module can effectively extract various types of features in images, and the attention module enables the extracted features to contain more abundant information. Skip connections are introduced to fuse different depth features to extract more effective features. Compared to other low-light image enhancement methods, our proposed network can extract more important features, thereby enhancing the quality of the low-light images.

### 3.2. Proposed Discriminator

**Figure 4:**
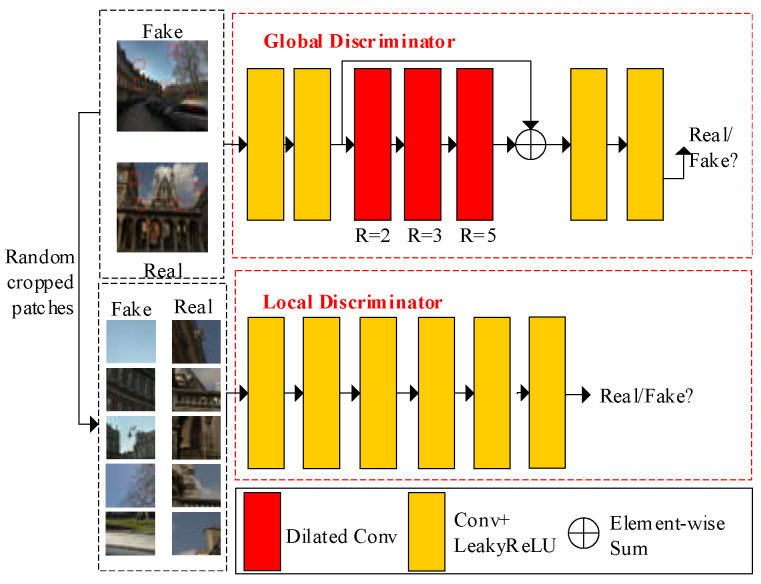
Proposed discriminator.

The discriminator is responsible for determining whether the input image is the enhancement image or the original normal-light image. It improves image recovery in generative networks by leveraging the interplay between the generator and discriminator. To improve the discriminatory ability of the discriminator, we designed the discriminator shown in Figure 4. This discriminator consists of a global discriminator and a local discriminator. The global discriminator consists of four standard convolutions with a LeakyReLU activation function and a residual module. The residual module comprises three cascaded 3 × 3 dilated convolutions with dilated rates of 2, 3, and 5. By utilizing dilated convolutions, the receptive field of the discriminator is increased without an increase in the number of parameters. This improvement in the discriminative ability is achieved by capturing more contextual information from the input image. Additionally, to mitigate pixel loss during the cascaded dilated convolution, a shortcut connection is introduced.

We used the patches obtained by randomly cropping the input image of the global discriminator as the input of the local discriminator. It can reduce the underexposure or overexposure of the enhanced image. Our global discriminator and local discriminator both adopt the Markovian discriminator structure, which can obtain more information in the image and improve the ability to discriminate between original high-quality images and generated high-quality images.

### 3.3. Proposed Loss Function

In order to improve the performance of low-light image enhancement, we proposed the improved loss function by introducing pixel loss. The complete loss function for our proposed generative adversarial networks is expressed as:

(7) $$L\mathrm{oss}=L_{GAN}^{Global}+L_{GAN}^{Local}++L_{Perceptual}^{}+L_{pixel}^{}$$

where $L_{GAN}^{Global}$ and $L_{GAN}^{Local}$ are the loss functions of the global discriminator and local discriminator that are shown in Figure 4, respectively. The $L_{GAN}^{Global}$ consists of two parts: $L_{D}^{Global}$ and $L_{G}^{Global}$. They are expressed as:

(8) $$L_{G}^{Global}=E_{z\sim p_{feak(z)}}{\left[D^{Global}(G(z))-1\right]}^{2}$$

(9) $$L_{D}^{Global}=E_{x\sim p_{real(x)}}{\left[(D^{Global}(x)-1)\right]}^{2}+E_{z\sim p_{feak(z)}}{\left[D^{Global}(G(z))\right]}^{2}$$

where G is the generator of generative adversarial networks. $D^{Global}$ is the global discriminator. x and z are the normal-light image and input low-light image of the generator, respectively. The $p_{real}(x)$ and $p_{fake}(z)$ are normal-light images and generated images by the generator, respectively. The $L_{GAN}^{Local}$ consists of two parts: $L_{D}^{Local}$ and $L_{G}^{Local}$. They are expressed as:

(10) $$L_{G}^{Local}=E_{z\sim p}{}_{{}_{{}_{fake}(z)}}{\left[D^{Local}(G(z))-1\right]}^{2}$$

(11) $$L_{D}^{Local}=E_{x\sim p_{real(x)}}{\left[(D^{Local}(x)-1)\right]}^{2}+E_{z\sim p_{feak(z)}}{\left[D^{Local}(G(z))\right]}^{2}$$

where $D^{Local}$ is the local discriminator. $p_{real}(x)$ and $p_{fake}(z)$ are normal light image patches and generated image patches by the generative network, respectively.

The perceptual loss is expressed as:

(12) $$L_{perceptual}(x)=\frac{1}{W_{i,j}H_{i,j}}\sum_{x=1}^{W_{i,j}}\sum_{y=1}^{H_{i,j}}||\varphi_{i,j}(G(x))-\varphi_{i,j}(x)|{|}_{2}^{2}$$

where x represents the original low-light image, G(x) represents the enhanced light image, and $\varphi_{i,j}$ represents the feature map obtained from the jth convolutional layer in the ith block of the pre-trained VGG16 network. $W_{i,j}$ and $H_{i,j}$ represents the feature map dimension. The pixel loss is used to measure the distance between the enhanced light image and the original low-light image. It is expressed as

(13) $$L_{pixel}=\sum_{i=1}^{m}{\left(x-G(x)\right)}^{2}$$

where x represents the original low-light image and G(x) represents the enhanced-light image.

## 4. Simulation and Discussion

In this section, we conduct a comparative analysis of our method with seven low-light enhancement methods: the Alpha-rooting method [26], the LIME method [27], the CycleGAN method [28], the Retinex-Net method [9], the EnlightenGAN method [22], the Zero-DCE method [11], and the Zero-DCE++ method [12]. We evaluated the performance of these methods on both a no-reference image dataset and a full-reference image dataset. We used NIQE (Natural Image Quality Evaluation) [29], SSIM (Structural Similarity) [30], PSNR (Peak Signal-to-Noise Ratio) [31], and BRISQUE (Blind/Referenceless Image Spatial Quality Evaluator) [32] as evaluation metrics. In our experiments, the batch size was 4, and other parameters were set to zero to initialize the network parameters. For a fair comparison, we used the same Adam optimizer and parameters $\beta_{1}=0.5$ and $\beta_{2}=0.999$ as used in EnlightenGAN [22] to optimize the loss function of the network. The network was trained with 200 epochs, a learning rate of 0.0001 for the first 100 epochs, and a linear decrease to 0 for the next 100 epochs. The whole training process is described in Algorithm 1. We used the Ubuntu 18.04 system in our experiment. The GPU is an NVIDIA GeForce GTX 2080Ti. The deep-learning framework is PyTorch.

**Algorithm 1.** Training procedure for our proposed method.1: **For**
*K* epochs **do** 2:    **For**
*k*(*k* is a hyperparameter, *k* = 1) steps **do** 3:     Sample minibatch of *m* low-light image samples {*z ^(1)^*,…, *z^(m)^*} from low-light image domain. 4:     Sample minibatch of *m* normal-light image samples {*z ^(1)^*,…, *z^(m)^*} from normal-light image domain. 5:     Update the discriminator by Adam Optimizer:$\nabla_{D}\left\{E{\left[(D(x^{\left(i\right)})-1)\right]}^{2}+E{\left[D(G(z^{\left(i\right)}))\right]}^{2}\right\}$6:     **End for** 7:   Sample minibatch of *m* low-light image samples { *z ^(1)^*,…, *z^(m)^*} fromlow-light image domain. 8:   Update the generator by Adam Optimizer:$\nabla_{G}\left\{E{\left[D(G(z^{\left(i\right)}))\right]}^{2}\right\}$9: **End for**

### 4.1. Datasets and Metrics

We used the dataset used in reference [22] as our training dataset. It has 914 low-light images and 1016 normal-light images with size 600 × 400. We selected 150 low-light images from the SICE dataset as the no-reference image test dataset [33]. We used the LOL dataset that includes 500 paired low-light/normal illuminated images as the full-reference image test dataset [9]. The Natural Image Quality Evaluation (NIQE), Structural Similarity (SSIM), Peak Signal-to-Noise Ratio (PSNR), and BRISQUE(Blind/Referenceless Image Spatial Quality Evaluator) are used to evaluate the performance of our method and the other seven methods. The NIQE is a no-reference evaluation metric. NIQE can be expressed as the distance between the MVG (Multivariate Gaussian) model of NSS (natural scene statistics) features extracted from the test image and the MVG model of perceptual quality features extracted from the natural image. It can be expressed as:

(14) $$NIQE=\sqrt{\left({\left(v_{1}-v_{2}\right)}^{T}{\left(\frac{\sum {}_{1}+\sum {}_{2}}{2}\right)}^{-1}(v_{1}-v_{2})\right)}$$

where $v_{1}$, $v_{2}$, $\sum {}_{1}$ and $\sum {}_{2}$ are denoted as the mean vector and covariance matrix of the MVG model for natural images and the MVG model for test images. The NIQE metric is used to evaluate the quality of the enhanced image, where a lower NIQE score indicates higher image quality. BRISQUE is a no-reference image quality assessment. It involves extracting mean subtracted contrast normalized coefficients (MSCN) from the test image, and fitting the MSCN values to an asymmetric generalized Gaussian distribution (AGGD). The features of the fitted Gaussian distribution are extracted and input to a support vector machine (SVM) for regression to obtain the assessment results of image quality. BRISQUE can be expressed as:

(15) $$\hat{F}\left(m,n\right)=\frac{F\left(m,n\right)-\mu_{g}\left(m,n\right)}{\sigma_{g}\left(m,n\right)+C}$$

where F(m,n) is the test image, $\hat{F}\left(m,n\right)$ is the mean subtracted contrast normalized coefficient at each pixel, $\mu_{g}\left(m,n\right)$ is the local mean signal value, and $\sigma_{g}\left(m,n\right)$ is the local contrast function. The smaller the NIQE, the higher the quality of the enhanced image. The SSIM is used to measure the similarity of two images, which can be expressed as:

(16) $$SSIM(x,y)=\frac{\left(2\mu_{x}\mu_{y}+c_{1})(2\sigma_{xy}+c_{2}\right)}{\left(\mu_{x}^{2}+\mu_{y}^{2}+c_{1})(\sigma_{x}^{2}+\sigma_{y}^{2}+c_{2}\right)}$$

where x and y represent the grayscale image of the enhanced image and the grayscale image of the normal-light image, respectively. $\mu_{x}$ and $\mu_{y}$ represents the mean value of x and y, respectively. $\sigma_{x}$ and $\sigma_{y}$ represents the variance of x and y, respectively. $\sigma_{xy}$ represents the covariance of x and y. $c_{1}={\left(0.01\times L\right)}^{2}$ and $c_{2}={\left(0.03\times L\right)}^{2}$ are the constant terms to maintain stability. L is the dynamic range of pixel values. The larger the SSIM value, the higher the quality of the enhanced image.

The PSNR is also used to measure the quality of the enhanced image. It can be expressed as

(17) $$PSNR=20\cdot \log_{10}(\frac{MAX_{I}}{\sqrt{MSE}})$$

where $MAX_{I}$ represents the maximum value of image pixel color and MSE represents the mean square error, which can be expressed as

(18) $$MSE=\frac{1}{mn}\sum_{i=0}^{m-1}\sum_{j=0}^{n-1}||I_{en}(i,j)-I_{gt}(i,j)|{|}^{2}$$

where $I_{en}$ and $I_{gt}$ represent the grayscale image of the enhanced image and the grayscale image of the normal-light image. The larger the PSNR, the higher quality of the enhanced image.

### 4.2. Ablation Study

In this section, we performed an ablation study to evaluate the performance of each module in our designed network. We compared our complete method with several variations, including our method without the attention module without parallel dilated convolution, without three cascaded dilated convolutions with residual structure, and without the pixel-loss function. The full-reference image dataset is used as a test dataset. The results are shown in Table 1. The PSNR values are 26.6451, 20.5918, 23.3964, 25.9701, and 26.1541 for our complete method, our method without attention module, our method without parallel dilated convolution, our method without cascaded dilated convolution, and our method without pixel-loss function, respectively. The SSIM values are 0.8817, 0.7686, 0.7761, 0.8667, and 0.8771 for our method and the other methods based on different modules, respectively. The NIQE values are 4.4719, 6.7748, 5.2070, 4.8803, and 4.9612 for our method and the other methods based on different modules, respectively. The BRISQUE values are 21.2218, 25.3088, 22.2576, 21.5149, and 40.0626 for our method and the other methods based on different modules, respectively. Our complete method with all modules has the largest values in PSNR and SSIM, and the smallest NIQE and BRISQUE. These results demonstrate that each module we proposed plays a crucial role in enhancing low-light images

### 4.3. No-Referenced Image Quality Assessment

**Figure 5:**
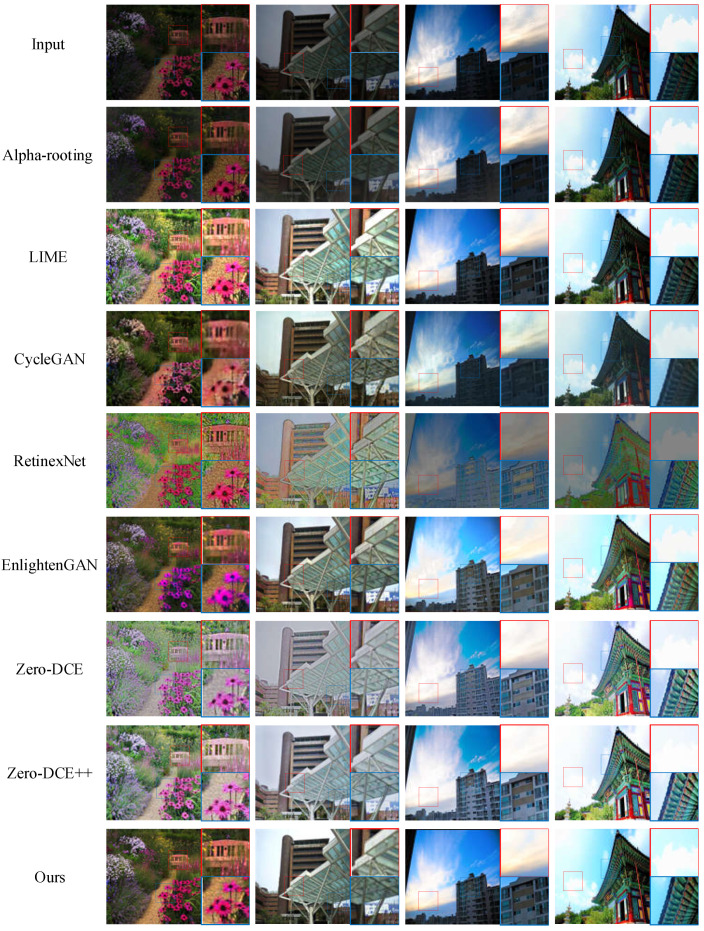
Low-light image enhancement results on the no-reference dataset.

We randomly selected two images from the no-reference dataset and two images that were previously used in the EnlightenGAN method as input images. We compared our proposed method with the Alpha-rooting method, the LIME method, the CycleGAN method, the Retinex-Net method, the EnlightenGAN method, the Zero-DCE method, and the Zero-DCE++ method. The low-light images and the enhanced images by different methods are shown in Figure 5. Each image contains a complete image and two enlarged detail images. The first row of images contains the original low-light images. The second to the eighth rows are the enhanced images by the Alpha-rooting method, LIME method, CycleGAN method, Retinex-Net method, EnlightenGAN method, Zero-DCE method, Zero-DCE++ method, and our proposed method, respectively.

In the first column, the images enhanced by the CycleGAN method and Retinex-Net method exhibit noticeable image blurring. The image enhanced by the Alpha-rooting method does not improve the brightness of the image. The image enhanced by the LIME method improves the brightness of the image but loses some details, which affects the visual perception. The image enhanced by the EnlightenGAN method shows color distortion. The images enhanced by the Zero-DCE method and the Zero-DCE++ method suffer from significant overexposure. In the second column, the images enhanced by the Alpha-rooting method exhibit noticeable image blurring. The image enhanced by the LIME method is overexposed. The image enhanced by the CycleGAN method appears underexposed and blurry. The image enhanced by the Retinex-Net method exhibits artifacts. The image enhanced by the EnlightenGAN method is underexposed. The images enhanced by Zero-DCE and Zero-DCE++ suffer from being overexposed. In the third and fourth columns, the LIME method and Alpha-rooting method do not effectively improve the brightness of the images. The images enhanced by the Retinex-Net method have better brightness, but the colors of the enhanced images are unnatural and affect visual perception. The images enhanced by the CycleGAN method appear noisy and more blurred. The enhanced images by the EnlightenGAN method show some color bias. The enhanced images by the Zero-DCE method and Zero-DCE++ method still exhibit overexposure.

In summary, the CycleGAN method generates low-quality enhanced images. The Alpha-rooting method does not effectively improve the brightness of the image. The LIME method often results in a loss of details in the enhanced images. The Retinex-Net method introduces color distortion and artifacts. The EnlightenGAN method suffers from color distortion. The Zero-DCE and Zero-DCE++ methods tend to overexpose the enhanced images. In comparison, our proposed method demonstrates superior performance. The images enhanced by our method exhibit better brightness and sharpness. They appear more natural and better visually for different low-light images.

The NIQE values of the four enhanced images by different methods are shown in Table 2. It is evident that the NIQE values for the 1st, 2nd, 3rd, and 4th enhanced images using our method are lower than those obtained by the other methods. Moreover, the average NIQE value of the enhanced images produced by our method is the smallest, followed by the EnlightenGAN method and the Zero-DCE++ method. Similarly, the BRISQUE values of the four enhanced images by different methods are shown in Table 3. The BRISQUE values for the 1st, 2nd, 3rd, and 4th enhanced images using our method are smaller than those of the other methods. Moreover, the average BRISQUE value of the enhanced images by our method is also the smallest, followed by the Zero-DCE++ method and the EnlightenGAN method. These results indicate that our proposed method outperforms other methods in enhancing low-light images.

To quantitatively analyze the performance of different methods, we utilized all images from the no-reference dataset as input low-light images of each method. The results are shown in Table 4. The average NIQE values are 8.8655, 8.9884, 8.8816, 7.8564, 5.2901, 6.1564, 5.7911, and 4.8656 for the Alpha-rooting method, LIME method, CycleGAN method, Retinex-Net method, EnlightenGAN method, Zero-DCE method, Zero-DCE++ method, and our proposed method, respectively. Furthermore, the average BRISQUE values are 41.0368, 40.3652, 39.6235, 41.3498, 30.2563, 28.6392, 24.3687, and 23.6987 for the Alpha-rooting method, LIME method, CycleGAN method, Retinex-Net method, EnlightenGAN method, Zero-DCE method, Zero-DCE++ method, and our proposed method, respectively. From the results, it can be observed that our proposed method has the smallest average NIQE and BRISQUE, followed by the EnlightenGAN method and Zero-DCE method. This shows that our proposed method outperforms other methods in enhancing low-light images based on these evaluation metrics.

### 4.4. Full-Referenced Image Quality Assessment

**Figure 6:**
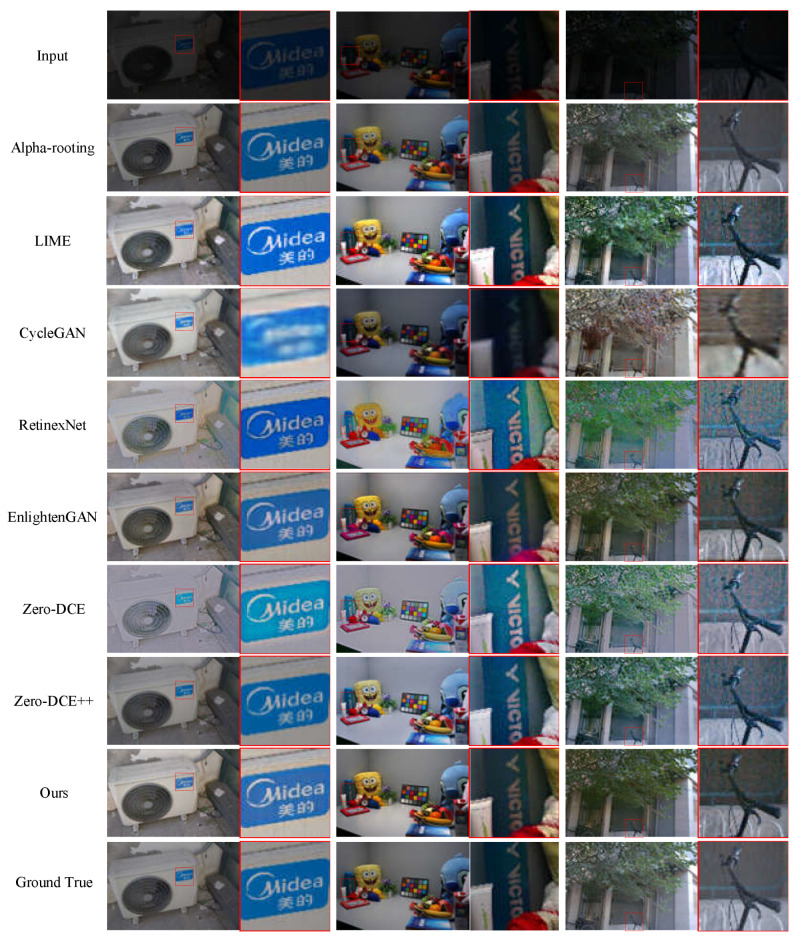
Low-light image enhancement results in the full-reference image test set.

We randomly selected two images from the no-reference dataset and one image that was previously used in the EnlightenGAN method as input images. We compared the LIME method, CycleGAN method, Retinex-Net method, EnlightenGAN method, Zero-DCE method, and Zero-DCE++ method with our proposed method. The low-light images and the enhanced images by different methods are shown in Figure 6. Each image contains a complete image and enlarged detail images. The first row shows the original low-light images. The second to the eighth rows display the enhanced images by the Alpha-rooting method, LIME method, CycleGAN method, Retinex-Net method, EnlightenGAN method, Zero-DCE method, Zero-DCE++ method, and our proposed method, respectively. The ninth row presents the original normal light images.

In the first column, the brightness of the images enhanced with the Alpha-rooting method is low. The images enhanced by the LIME method and the EnlightenGAN method have better brightness, but the LIME method results in a loss of detailed information, while the EnlightenGAN method produces underexposed images. The images enhanced by the CycleGAN method have poor clarity. The image enhanced by the Retinex-Net method shows obvious color distortion. The images enhanced by the Zero-DCE method and Zero-DCE++ method suffer from overexposure. In the second column, the image enhanced by the LIME method contains noise, such as numerous spots in the enlarged part of the letters, which negatively affects human visual perception. The image enhanced using the CycleGAN method exhibits blurring artifacts. The image enhanced using the Alpha-rooting method appears to be underexposed. The images enhanced by the EnlightenGAN method and Retinex-Net exhibit color distortion, and the Retinex-Net also introduces artifacts. The images enhanced by the Zero-DCE method and Zero-DCE++ method still suffer from overexposure. In the third column, the image enhanced by the Alpha-rooting method shows obvious color distortion. The image enhanced by the CycleGAN method has low sharpness and severe color distortion. The image enhanced by the LIME method contains a lot of noise. The images enhanced by the Retinex-Net method and EnlightenGAN contain color distortion. The image enhanced by the Zero-DCE method and Zero-DCE++ still suffers from overexposure.

On the whole, the CycleGAN method has the worst image enhancement performance, resulting in images with high noise levels and low sharpness. The Alpha-rooting method does not improve the brightness of the image, and the color of the image enhanced by this method appears distorted. The EnlightenGAN method and LIME method exhibit better performance in image enhancement. However, the EnlightenGAN generates color distortion, while the LIME method generates significant noise. The images enhanced by the Retinex-Net method have high brightness but also have a lot of speckles. The Zero-DCE method and Zero-DCE++ method are prone to overexposure. In contrast, the images enhanced by our proposed method contain a large amount of image information while having better visual quality and better matching the real-light images.

The values of PSNR, SSIM, NIQE, and BRISQUE for three enhanced images by different methods are shown in Table 5. For the same low illumination images, each image enhanced with our proposed method has the largest SSIM and PSNR values as well as the smallest NIQE and BRISQUE values. These results show that the quality of the enhanced image by our method is better than the enhanced image by other methods.

We conducted quantitative experiments to further verify the superiority of our method’s performance. We tested different methods using all the images in the full-reference dataset, and the corresponding values of PSNR, SSIM, NIQE, and BRISQUE are presented in Table 6. The average PSNR values are 17.3354, 17.8337, 21.1463, 17.7947, 23.9674, 19.7008, 18.8698, and 26.6451 for the Alpha-rooting method, LIME method, CycleGAN method, Retinex-Net method, EnlightenGAN method, Zero-DCE method, Zero-DCE++ method, and our proposed method, respectively. The proposed method achieves the highest PSNR, followed by EnlightenGAN and CycleGAN. The average SSIM values are 0.7022, 0.6321, 0.8322, 0.6257, 0.8640, 0.7416, 0.6463, and 0.8817 for the Alpha-rooting method, LIME method, CycleGAN method, Retinex-Net method, EnlightenGAN method, Zero-DCE method, Zero-DCE++ method, and our proposed method, respectively. The proposed method has the highest SSIM, followed by EnlightenGAN and CycleGAN. The average NIQE values are 8.9621, 8.9673, 8.1960, 6.9928, 4.8963, 6.0023, 5.3725, and 4.4719 for the Alpha-rooting method, LIME method, CycleGAN method, Retinex-Net method, EnlightenGAN method, Zero-DCE method, d Zero-DCE++ method, and our proposed method, respectively. The proposed method has the highest PSNR, followed by EnlightenGAN and Zero-DCE++. The average values of BRISQUE are 39.6477, 40.3188, 30.3485, 34.5698, 23.2056, 29.4853, 24.8423, and 21.2218 for the Alpha-rooting method, LIME method, CycleGAN method, Retinex-Net method, EnlightenGAN method, Zero-DCE method, Zero-DCE++ method, and our proposed method, respectively. The proposed method has the highest PSNR, followed by EnlightenGAN and Zero-DCE++. Based on Table 6, our proposed method has larger PSNR and SSIM values than other methods, and our proposed method has smaller NIQE and BRISQUE values than other methods. This confirms that our proposed method outperforms other methods in enhancing low-light images.

## 5. Conclusions

This paper introduces a novel generative adversarial network for unsupervised low-light image enhancement. The generator adopts the residual module, hybrid attention module, parallel dilated convolution module, and skip connection, which can effectively extract high-frequency information and low-frequency information in the input image. The discriminator in our approach incorporates a global-local discriminator that is based on the principles of a Markovian discriminative network. This design choice allows our network to capture both global and local image characteristics effectively. To further enhance the discriminative capabilities of the global discriminator, we introduce three cascaded dilated convolutions to increase the receptive field. To further improve the enhancement performance, the paper introduces an improved loss function that integrates pixel loss into the loss function of the generative adversarial network. This addition helps in recovering more detailed information in the enhanced images. The proposed method is evaluated on both full-reference and no-reference datasets, and comprehensive experimental results demonstrate its superior performance compared to seven existing methods for low-light image enhancement.

## Figures and Tables

**Table 1:** Assessment results of each module.

	No_Attention	No_Parallel Dilated Conv	No_Cascaded Dilated Conv	No_Pixel Loss	Ours
PSNR	20.5918	23.3964	25.9701	26.1541	26.6451
SSIM	0.7686	0.7761	0.8667	0.8771	0.8817
NIQE	6.7748	5.2070	4.8803	4.9612	4.4719
BRISQUE	25.3088	22.2576	21.5149	40.0626	21.2218

**Table 2:** NIQE values of four enhanced images by different methods.

	Alpha	LIME	CycleGAN	Retinex-Net	EnlightenGAN	Zero-DCE	Zero-DCE++	Ours
1st image	6.9561	6.6348	7.5064	12.4557	5.2253	8.8149	7.8973	5.1815
2nd image	7.1162	4.7842	5.8236	6.7309	3.6692	5.1637	4.5945	3.4426
3rd image	8.2354	8.3674	6.5934	10.6873	5.2263	6.3784	6.1283	5.1637
4th image	8.3671	6.3724	7.6354	11.3648	4.6992	4.4762	4.1164	3.9651
Average	7.6687	6.5397	6.8897	10.3097	4.7050	6.2083	5.6841	4.4382

**Table 3:** BRISQUE values of four enhanced images by different methods.

	Alpha	LIME	CycleGAN	Retinex-Net	EnlightenGAN	Zero-DCE	Zero-DCE++	Ours
1st image	42.6327	43.6249	38.6523	44.2361	30.4125	36.2578	33.6245	30.5261
2ndimage	41.2961	40.4375	40.1263	48.2763	29.1547	30.1542	28.7163	20.5238
3rd image	36.1284	37.9658	28.1267	38.1476	23.6321	29.3697	24.3698	22.9086
4th image	39.6183	50.5563	36.2548	53.2174	35.2147	40.1236	29.7211	25.4236
Average	39.9189	43.1461	35.7900	45.9694	29.6035	33.9763	29.1079	24.8455

**Table 4:** Average NIQE and BRISQUE values of enhanced images by different methods on a no-reference dataset.

	Alpha	LIME	CycleGAN	Retinex-Net	EnlightenGAN	Zero-DCE	Zero-DCE++	Ours
NIQE	8.8655	8.9884	8.8816	7.8564	5.2901	6.1564	5.7911	4.8656
BRISQUE	41.0368	40.3652	39.6235	41.3498	30.2563	28.6392	24.3687	23.6987

**Table 5:** Performance index of three images enhanced by different methods.

		Alpha	LIME	CycleGAN	Retinex-Net	EnlightenGAN	Zero-DCE	Zero-DCE++	Ours
1st image	PSNR	20.1381	19.8378	23.0964	15.9278	21.8685	15.9880	15.4431	24.1329
	SSIM	0.8032	0.7732	0.7944	0.7839	0.9213	0.8529	0.6098	0.9267
	NIQE	5.9932	6.9635	7.2511	7.5961	4.9968	5.0166	4.9888	4.7602
	BRISQUE	36.5827	34.5062	36.7823	28.1026	23.6384	26.8221	24.7335	23.0285
2nd image	PSNR	19.6382	17.9721	19.8103	13.2096	23.2018	16.5817	16.3813	27.3032
	SSIM	0.7886	0.7770	0.8515	0.7312	0.9208	0.8294	0.7320	0.9352
	NIQE	7.6631	8.3652	8.2236	6.2358	3.9624	4.9968	4.6379	3.8891
	BRISQUE	37.8260	36.5896	31.2014	24.1036	28.0457	28.0211	27.4125	24.1027
3rd image	PSNR	18.6357	17.3886	19.0584	15.8160	17.3886	17.2072	18.8322	20.5577
	SSIM	0.7081	0.6572	0.6837	0.6637	0.8802	0.7673	0.6311	0.8695
	NIQE	9.6635	7.6354	9.6102	5.1632	5.1022	5.8906	5.1063	4.9924
	BRISQUE	31.2569	30.1265	29.6321	29.3678	30.9519	37.2673	36.1859	28.6571
Average	PSNR	19.3764	18.3995	22.6550	14.9845	20.8196	16.5923	16.8855	23.9979
	SSIM	0.7815	0.7358	0.7765	0.7263	0.9074	0.8165	0.6576	0.9101
	NIQE	8.9657	7.6547	8.3616	6.3317	4.6871	5.3013	4.9110	4.5472
	BRISQUE	34.5632	33.7408	32.5386	29.1913	27.5453	30.7035	29.4440	25.2628

**Table 6:** Performance index of all images enhanced by different methods.

	**A** **lpha**	**LIME**	**CycleGAN**	**Retinex-Net**	**EnlightenGAN**	**Zero-DCE**	**Zero-DCE++**	**Ours**
PSNR	17.3354	17.8337	21.1463	17.7947	23.9674	19.7008	18.8698	26.6451
SSIM	0.7022	0.6321	0.8322	0.6257	0.8640	0.7416	0.6463	0.8817
NIQE	8.9621	8.9673	8.1960	6.9928	4.8963	6.0023	5.3725	4.4719
BRISQUE	39.6477	40.3188	30.3485	34.5698	23.2056	29.4853	24.8423	21.2218

## Data Availability

Not applicable.
